# Pertussis Notification Rate and Tdpa Vaccine/Booster Coverage in Adults: An Opportunity for an Epidemiological Observatory in Primary Care

**DOI:** 10.3390/idr16050068

**Published:** 2024-09-02

**Authors:** Francesco Lapi, Ettore Marconi, Iacopo Cricelli, Alessandro Rossi, Tecla Mastronuzzi, Giovanni Gabutti, Claudio Cricelli

**Affiliations:** 1 Health Search, Italian College of General Practitioners and Primary Care, Via del Sansovino 179, 50142 Florence, Italy; marconi.ettore@simg.it; 2 Genomedics SRL, 50141 Florence, Italy; iacopo.cricelli@genomedics.it; 3 Italian College of General Practitioners and Primary Care, 50142 Florence, Italy; rossi.alessandro@simg.it (A.R.); mastronuzzi.tecla@simg.it (T.M.); cricelli@gmail.com (C.C.); 4 Working Group “Vaccines and Immunization Policies” of the Italian Scientific Society of Hygiene, Preventive Medicine and Public Health (SItI), Cogorno, 16030 Genoa, Italy; gbtgnn@unife.it

**Keywords:** pertussis, dTap, observatory, primary care

## Abstract

**Background**: In recent years, Europe has experienced a significant increase in pertussis cases. One reason behind this rise is the decline in diphtheria–tetanus–pertussis (dTap) booster coverage among adults. Currently, Italy lacks a reliable monitoring system to track pertussis infections and vaccine coverage among adults. We therefore evaluated the reliability of a primary care framework to respond to this need. **Methods**: Using an Italian primary care database for individuals aged 15 or above, we determined the pertussis infection notification rate and dTap vaccine/booster coverage for the timeframe of 2009–2022. **Results**: In the overall population, we obtained a lifetime occurrence rate of pertussis infections of 7.52 per 10,000 individuals. The annual incidence rates of pertussis infections ranged from 0.008 to 0.001 per 10,000 person-years between 2009 and 2022. A rising trend in dTap vaccine coverage rate (ranging from 8.72 to 16.54 vaccines per 10,000 individuals) was observed during the same period. Notably, those aged 65 or older, smokers, and/or individuals with immunodeficiencies were more likely to receive the dTap vaccine compared to the general population. **Conclusions**: Given the organization of the Italian public health system, this primary care network might act as a reliable epidemiological monitoring system to keep track of pertussis infections and dTap vaccine coverage in adults. Pertussis cases were underreported, and there was a low uptake of vaccines and boosters. Therefore, it is crucial to closely monitor pertussis notifications and dTap administrations and develop intervention strategies at the national level to enhance vaccine-related prevention.

## 1. Background

Pertussis is a respiratory infectious disease caused by *Bordetella pertussis*, a Gram-negative aerobic non-spore-forming bacterium. This infection is highly contagious with a reported R0 value between 12~17. It is mainly transmitted via droplets, with outbreaks usually every 3–5 years in the pre-immunization era (Summer–Autumn seasonality) [1]. In the pre-immunization and pre-antibiotic era, the incidence and fatality rates due to this infection are very high, especially among children aged 5 years or younger. In the US, after the introduction of immunization in the 40s, the incidence rates significantly declined, moving from 150 cases to 6.1 cases per 100,000 people being registered in 2011. Infants younger than 1 year reported the highest incidence rate with 126.7 cases per 100,000 people [2]. A US analysis of a 5-year period of pertussis surveillance suggested that highly vulnerable patients might show a greater risk for severe pertussis infections; namely, 27% of patients aged <1 year and 12% of patients aged ≥65 years were hospitalized for their pertussis infection [3].

In 2022, there were 2623 reported cases of pertussis in Europe, with a notification rate of 0.7 cases per 100,000 inhabitants. This rate was slightly higher compared to the previous year, following a significant decrease in cases during the COVID-19 pandemic in 2019 and 2020. Infants under one year of age were the most impacted age group, with the highest notification rate at 4.0 per 100,000 people. Surprisingly, nearly 70% of all cases were reported in individuals over 15 years of age [4]. Along this line, in spite of the proven effectiveness of the diphtheria–tetanus–pertussis vaccine (i.e., DTaP and dTap for children and adults, respectively) [5], a new increase in pertussis cases has been registered in several countries since 2016, suggesting that adults might play an important role in the new dynamics of the infection [6,7]. In Italy, a notification rate of 0.28 per 100,000 people in those aged 15 years or older is therefore underestimated [8]. More recently, the ECDC reported two further peaks of 25,130 cases of pertussis in 2023 and more than 30,000 between January and March 2024. Among the 19 fatal cases registered, 11 (58%) were seen in infants and 8 (42%) in those aged 60 years or older. These two new waves of infections were similar to those observed in 2016 [4]. Such evidence is likely due to the reduction in dTap booster administration in adults being observed in recent decades, which contributed to the changes in epidemiology of pertussis infections as immunity wanes [6,7,9]. Furthermore, there are limitations in the surveillance system as well, for both monitoring notifications of pertussis infections and dTap vaccine coverage in adults. While in the pediatric population the presence of pertussis is more easily diagnosed and notified and vaccine coverage is close (>95%) to be optimal [5], a reliable epidemiological observatory on pertussis for adults is still lacking. In this context, individuals aged 65 years or older, those with asthma and/or COPD, and/or immunodeficient people are more likely to experience higher rates of pertussis-related hospitalizations and healthcare costs, as well as serve as a potential source of infection for susceptible children, according to the available evidence [10,11].

Taking into consideration the aforementioned context, General Practitioners (GPs), who are the first point of contact for patients with this condition, have a crucial part to play in identifying unreported cases of pertussis and monitoring vaccinations. In this regard, the Health Search Database (HSD), which is comparable to the Italian population based on the Italian Official Statistics (ISTAT) [10,12], could be the ideal tool to provide population-based surveillance of pertussis in terms of the notification rate of infections and the monitoring of vaccine administrations. To this end, we quantified the notification rate and vaccine coverage of pertussis among adults registered in this data source.

## 2. Methods

### 2.1. Data Source

This analysis was conducted using the Health Search Database (HSD) of the Italian College of General Practitioners (GPs) and Primary Care. The HSD is a longitudinal observational database established in 1998 by the Italian College of GPs, containing the electronic patient records from approximately 1000 GPs throughout Italy. Computer-based patient records collected by a selected group of 800 GPs, who met standard quality criteria regarding the levels of data entry (i.e., levels of coding, prevalence of selected diseases, rates of mortality, and years of recording), were included in the present analysis. These GPs covered about 1 million patients and were selected on a geographical basis to include patients being representative of the whole Italian population and to ensure the completeness and consistency of medical records. They consisted of demographic details; medical information, such as diagnoses, drugs, and diagnostic test prescriptions; specialist referrals; life-style characteristics; and mortality. All these data were linked through a unique anonymous individual identification number. All diagnoses were coded according to the International Classification of Diseases, revision 9, Clinical Modification (ICD-9-CM). To complement the coded diagnoses, GPs were able to add free text. Information on drug prescriptions included the name of the prescribed drug (i.e., active substance and/or brand name), the correspondent Anatomical Therapeutic Chemical (ATC), the Defined Daily Dose (DDD), the date of prescription, dosage instructions, and number of days’ supply. The ATC/DDD is a validated classification system from the World Health Organization (WHO). The scientific validity of the HSD has been demonstrated elsewhere [11,13,14].

### 2.2. Study Population

To evaluate the notification rate of pertussis infections, we formed a cohort of patients, aged 15 years or older, being registered in the HSD for January 2009 to 31 December 2022. The date of the first visit with their GP in the eligibility period was the study index date. The selected patients were followed up until the occurrence of the following events, whichever came first: occurrence of pertussis infection (i.e., event date: see Section 2.3 Outcome definition paragraph), death, end of data availability with patient’s GP, end of the study period (31 December 2022).

To evaluate the vaccine coverage, the study population included patients, aged 15 years or older, who were active (i.e., alive and currently registered in HSD) at the end of each year under investigation (2009–2022). For this analysis, the 31st December of each year was the study index date [11,13,15,16].

### 2.3. Outcome Definition

The pertussis diagnoses were identified via ICD9CM (0.33* combined with term “pertussis” in code description) and/or the presence of IgG and/or IgM for pertussis during the 10-year analysis period, on the basis of the examined measure. Namely, the lifetime prevalence of pertussis was calculated counting the “old” and “new” cases (presence of ICD9CM or IgG/IgM positive) of pertussis in the overall population, being active in the database on 31 December 2022. Instead, the yearly incidence density (i.e., the denominators formed by cumulated person-times) of pertussis was calculated over the entire analysis period by capturing the ICD9CM code of the infection or IgM positive (not IgG, given the aim to capture incident cases) being newly (with no prior diagnosis or IgM/IgG positive test) registered during follow-up.

The yearly vaccine coverage was operationally defined as registration of pertussis vaccination in the 10-year period preceding the index date [5]. The incidence densities were stratified for the presence of dTap vaccination. In addition, estimates of vaccine coverage were reported for those subjects active in the HSD on 31 December 2022 and featured clinical conditions predisposing people to pertussis-related complications. They comprised an age ≥ 65 years; a diagnosis of asthma (ICD9CM: 493*; in the entire period preceding or on vaccination date); COPD (ICD9CM: 496*; 491.2; in the entire period preceding or on vaccination); obesity (BMI ≥ 30 kg/m^2^ or ICD9CM: 278.0); current smoker (during the specific year in which pertussis diagnosis/vaccination was coded); and the presence of immunosuppressive condition (see Appendix A; in the entire period preceding or 1-month following the pertussis diagnosis/vaccination) [5,17].

### 2.4. Data Analyses

Continuous and categorical variables were reported as mean (SD) and proportional values, respectively. The lifetime prevalence (old (prior) and new cases) and incidence (new cases) density were reported with 95% Confidence Intervals (CIs) on yearly bases as well as on patients active on 31 December 2022. The incidence density was calculated by considering person-times as a denominator. All the analyses were stratified according to age and gender accordingly.

The incidence densities of pertussis were calculated for both unvaccinated and vaccinated subjects, nominally those who received the dTap injection within the past ten years. Furthermore, the proportions of vaccine coverage were compared using a *Χ*^2^ test for those with the aforementioned conditions [5], which are known risk factors for pertussis-related complications, vs. those reported in the unvaccinated population.

## 3. Results

**Figure 1:**
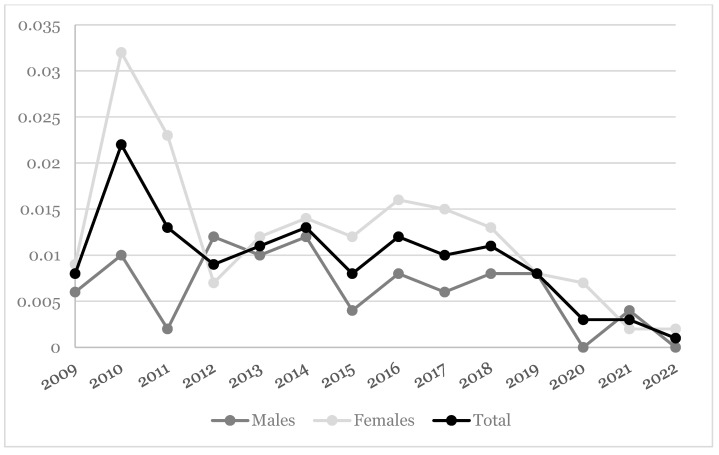
Incidence rate (per 10,000 person-years) of pertussis infections over the 14-year study period.

The population active in the database on 31 December 2022 was formed by 1,037,693 patients (52% females; mean age = 53.2, SD: 12.1; yearly denominators are reported in Supplementary Table S1). Table 1 depicts the lifetime prevalence of pertussis in the HSD. In the overall population, 780 patients were diagnosed with pertussis at least once in their life, corresponding to 7.52 per 10,000 individuals. Three fatal cases were registered. Females showed higher lifetime prevalence rates than males (8.6 vs. 6.5 per 10,000), and most of the cases were registered for younger individuals, especially those aged 25–34 years (lifetime prevalence equal to 23.8 per 10,000). Specifically, 659 cases of pertussis were identified via ICD9CM only, 121 were IgG and/or IgM positive for pertussis only, and 17 cases presented both conditions. The yearly incidence densities of pertussis are reported in Figure 1. Moving from 2009 to 2022, the incidence density range was 0.008–0.001 per 10,000 person-years. A steep increase was observed in 2010 and 2012 (0.022 and 0.013 per 10,000 person-years), which was mainly due to female subjects. After this, there has been a stable trend over the years under study since 2012. Females showed a higher incidence density than males over the years, with the exception of 2012.

**Figure 2:**
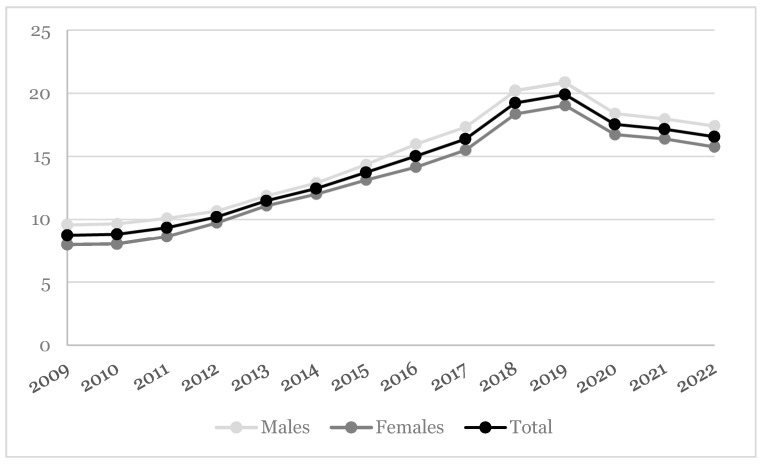
Vaccine coverage (per 10,000 person-years) over the 14-year study period.

For what concerns the vaccine coverage, we observed a growing trend for dTap coverage rate (i.e., injected/registered in the 10 years ascertaining the likely presence of immunity) ranging 8.72–16.5 vaccinees per 10,000 individuals between 2009 and 2022. The vaccine coverage among males was a little higher than females (Figure 2). When the incidence densities of notified cases of pertussis were stratified by vaccine coverage in the last 10 years, the pertussis infections were all registered in the unvaccinated patients. Only one case was reported among vaccinated patients. Among subjects active in the database on 31 December 2022, 1669 had at least 1 of the risk factors for disease progression (29.5 per 10,000). When compared with unvaccinated subjects, a higher significant vaccine coverage was reported for those aged 65 years or older (21.6 per 10,000; *p* < 0.001), followed by smokers (36.2 per 10,000; *p* < 0.001) and immunocompromised individuals (24.2 per 10,000; *p* = 0.008) (Table 2).

## 4. Discussion

To our knowledge, this is the first study to assess the current epidemiology of pertussis and dTap vaccination/booster coverage in an adult population in a primary care setting. These findings confirm the under-notification of pertussis infections and the poor coverage for the dTap vaccine/booster. This GP network could represent a reliable epidemiological observatory to monitor the epidemic phases of pertussis along with prevention strategies including vaccination campaigns. As stated in the background of this study, while in the pediatric setting there is an effective surveillance system, a similar framework is still lacking in general practice [18,19,20].

For what concerns the incidence densities of pertussis infections, our results were consistent with those reported in previous works. Brosio and coworkers [21] reported and incidence rate of pertussis-related hospitalizations with a range of >1–0.64 cases per 100,000 inhabitants between 2000 and 2014; along this line, the yearly estimates for pertussis notifications were similar to those reported by ECDC [8] between 2008 and 2018. Nevertheless, these findings were based on administrative data in which severe cases of pertussis are coded, namely those leading to hospital admission. In a GPs’ observatory, the notified cases might include milder cases, as well as those diagnosed in adults which are featured by atypical presentations [17,22], being carriers for susceptible children [6,7].

A recent study showed that the reported incidence rate of pertussis infections was generally low among adults aged 50–64 years (1–2 per 100,000) but seroprevalence surveys indicated a 4-fold underestimate of pertussis infections, demonstrating a higher burden of under notification for this age category [23]. As per Marchi and coworkers [24], since 2002, approximately half of the population over 22 years of age have low IgG titers. Interestingly, in 2013–2016, almost one third of subjects aged 12–22 years, nominally the age group most likely to have been vaccinated against pertussis in infancy, had low levels of antibodies. They were therefore presumably susceptible to acquiring and transmitting pertussis because of immunity waning. Recently, a sero-epidemiological study of *Bordetella pertussis* infection in the Italian general population has been published highlighting the possible role of adolescents and adults in the transmission dynamics of this pathogen [25]. The estimated rate of underreporting of pertussis infection based on this seroprevalence study was approximately 141-fold and 3452-fold higher than the notified incidence in the 6–14 and >15 age groups, respectively [26].

Thus, our incidence density of 0.11 per 100,000 in those aged 15 years or older is largely underestimated, as consistently demonstrated by ECDC data [4,8]. Not surprisingly, Leong and coworkers [27], using an Australian primary care data source with different operational definitions of pertussis-related GPs encounters, showed that general practice is able to provide a comprehensive estimate of this infection. Indeed, a more inclusive definition of pertussis-related encounters led to 64% higher incidence rate than national notification data. This is further evidence on the utility of primary care data, including ours, in providing a better understanding of pertussis-related epidemics in adults, in which its atypical presentation leads to challenges in diagnosis and a lack of awareness among public health providers [20,28,29,30]. In this respect, it is worth mentioning that pertussis was one of the most frequent cause of hospitalization in those aged ≤1 years [3,21,31], but it was not negligible in older adults who accounted for more than 10% of hospitalizations [3,22].

In Italy, according to the expected reduction in pertussis-related immunity, the booster vaccination is recommended every 10 years in subjects aged 65 years or older as well as those suffering from respiratory disease (asthma and/or COPD), obesity, smokers, and/or immunodepressive disorders, nominally those more prone to develop infection-related complications [5]. Nevertheless, dTap coverage data in adults are similarly scarce or inconsistent across Europe [9]. In Italy, a seroprevalence study performed in 2015 showed a dTap vaccination coverage among adults of only 38% [29]. More recently, Lecce and coworkers [32] compared the coverage rate with dTap requirements in 2019 across Italian regions, for a population aged 19 years or older. The national vaccine coverage rate was equal to 10.6% in the overall cohort and reached 75% for those aged 65 years or older. In addition, a relevant heterogeneity was observed across Italian regions. This investigation therefore confirmed the low vaccine coverage for dTap in adults, but it was not based on actual administrations.

Our data on dTap injections were therefore underestimated because of several reasons. First, the dTap vaccination programs differ across Italian regions, in which GPs are not always directly involved in vaccine administration. Second, GPs have the mandate to register vaccinations twice in the public regional registry and in their own electronic health records. Such an increase in GPs’ workload might therefore reduce the completeness of data collection. The same statements can be provided for what concerns fatal cases, which cannot be adequately reported without a reliable surveillance system.

This study has some limitations. First, the poor notification of cases of pertussis which led to underestimation of cumulative prevalence and incidence density. Nevertheless, among the study objectives, we aimed to underline this concern along with the absence of a systematic registration of vaccine coverage in adults, as stated by the recent ECDC report [4]. Second, the operational definition of pertussis might be prone to some misclassification, given that the presence of IgM was poorly registered as well [22,33]. However, given the clinical presentation (e.g., whooping cough mainly during night and emesis) [17] of this condition, and the fact that our estimates were consistent with prior works, the presence of false positives should likely be reduced. Third, although the actual vaccination coverage is expectedly low, we cannot rule out the possibility of a decreased registration of dTap vaccines in GPs’ records. This same rationale could be provided for the reduced coverage seen in patients with COPD, which appears unexpectedly low for this category. Nevertheless, the statistically significant relationships we found are likely to maintain their direction, given their consistency across high-risk patients who are susceptible to pertussis-related complications. Finally, we were unable to capture information on patients’ socioeconomic status which can be useful to comprehend and overcome the barrier to vaccine administration. Once more, GPs are likely to be the healthcare professionals who are closest to their patients, enabling them to more effectively comprehend the impact of these variables on vaccination rates [34].

Overall, the present findings indicate that reliable observations in the adult population are still needed to monitor every action implemented to increase pertussis identification and dTap vaccine coverage. In Italy, only the primary care setting is able to cover this role given the fact that the Italian public health system entitles every citizen to have a GP. Indeed, it has been demonstrated that vaccination rate was positively associated with increased GP/doctor visits, increased contacts with primary care, or having a regular consult with a GP [34]. Primary care surveillance would therefore complement a “call to action” [19] to better identify those patients’ categories, which, in light of conditions predisposing one to pertussis infections and/or its related complications, might majorly benefit from dTap immunization and limit disease transmission [30].

## 5. Conclusions

In Italy, as in other European countries, there is a new epidemiological phase of pertussis. As demonstrated by our data, a representative GP network might constitute a reliable framework to improve the surveillance of pertussis epidemics along with dTap vaccine coverage in adults. This surveillance system should aid public health providers to evaluate and offset the best immunization strategies, which are generally planned and implemented locally with different involvement of GPs. As such, transmission and pertussis-related morbidity and mortality might be effectively contained.

## Figures and Tables

**Table 1:** Lifetime prevalence, per 10,000 person-years, of pertussis infection in active population being registered in HSD on 31 December 2022.

	Males		Females		Total	
	N *	10,000	N *	10,000	N *	10,000
Age category						
15–24	30	5.81	43	9.51	73	7.54
25–34	165	22.19	178	25.62	343	23.85
35–44	54	6.86	84	11.09	138	8.94
45–54	32	3.31	57	5.97	89	4.63
55–64	33	3.49	47	5.12	80	4.29
65–74	10	1.44	14	2.20	24	1.81
75–84	11	2.74	15	2.97	26	2.87
≥85	2	1.39	5	1.92	7	1.73
TOTAL	337	6.48	443	8.56	780 *	7.52

N = pertussis cases; * = using ICD9CM codes and/or IgG/IgM pertussis-related tests.

**Table 2:** dTap vaccination/booster coverage (per 10,000 person-years) according to concurrent condition predisposing to pertussis-related complications in active population being registered in HSD on 31 December 2022.

	N	* 10,000	95% CI	*p* Value *
age ≥ 65 years	554	20.99	(19.28–22.81)	<0.001
asthma	272	30.36	(26.87–34.19)	0.094
COPD	83	29.83	(23.77–36.97)	0.29
obesity	1010	34.64	(32.54–36.84)	0.19
smoking	499	36.16	(33.06–39.47)	<0.001
immunodepressive disorders	64	24.17	(18.62–30.85)	0.008
at least one condition	1669	30.05	(28.63–31.53)	

* Chi-square test (vaccinated vs. unvaccinated subjects).

## Data Availability

Our data cannot be shared according to the database owner roles.

## Supplementary Materials

The following supporting information can be downloaded at: https://www.mdpi.com/article/10.3390/idr16050068/s1, Supplementary Table S1: Study population by year.

## Appendix A. Codes (with Italian Descriptions) for Immunosuppressive Diseases (Use of Related Medications Is Included)

Immunodepressive disorders

ICD9CM, disease:

279.0*—Deficit immunità umorale

279.1*—Deficit dell’immunita’ cellulare

279.2*—Deficit immunitario complesso

279.3*—Deficit immunitario

279.8*—Altri disordini specificati che riguardano i meccanismi immunitari

V08—Stato infettivo asintomatico da virus da immunodeficienza umana, HIV

279.2—aplasia o displasia timica con immunodeficienza 042—Infezione da virus immunodeficienza umana, HIV

279.2—Grave immunodeficienza combinata, SCID

334.8—Atassia—Telangiectasia, sindrome Louis-Bar

288.1—Disturbi funzionali polimorfonucleati neutrofili

288.2—Anomalie genetiche leucociti (Sindrome di Chédiak-Higashi)

284.0—Anemia aplastica costituzionale

284.8—Altre anemia aplastiche specificate

284.9—Anemia aplastica

204.1—Leucemia linfoide cronica

238.7—Sindrome mileodisplastica

273.3—Macroglobulinemia Waldenstrom

V42.0—Trapianto di rene

V42.1—Trapianto di cuore

V42.6—Trapianto di polmone

V42.7—Trapianto di fegato

V42.8—Trapianto di midollo/pancreas/intestino/

579.8—Linfangectasia intestinale

759.89—Sindrome di Proteo

758.6—Sindrome Turner

758.89—Sindrome di Noonan/klippel-trenaunay

359.2—Distrofia miotonica

**ATC, medications:**

L01XX32 Bortezomib

L04AA06 Micofenolato

L01AA01 Ciclofosfamide

L01AA02 Clorambucile

L01AA03 Melfalan

A07EC01 Sulfasalazina

L01BA01 Metotrexato

N05AH02 Clozapina

L01XE* inibitori protein chinasi L01BB05 Fludarabina L04AX01 Azatioprina L01XC02 Rituzimab

L01XC10 Ofatumumab L01XC15 Obinutuzumab L04AD01 Ciclosporina L04AD02 Tacrolimus L04AA10 Sirolimus

L01XC19 Blinatumomab

L04AA34 Alemtuzumab L04AA26 Belimumab

L04AA* immunosoppressori selettivi

L01XC06

L01XC03, Cetuximab

L01XC14 Trastuzumab

L04AC05 Ustekinumab

L04AB04 Adalimumab

L03AB* Interferoni

L04AB01 Etanercept

L01CA02 Vincristina

L01BC01 Citarabina

L04AB05 Certolizumab L04AB06 Golimumab

L04AC03 Anakinra

L04AA23 Natalizumab
