# Supplementary material for: Pertussis Notification Rate and Tdpa Vaccine/Booster Coverage in Adults: An Opportunity for an Epidemiological Observatory in Primary Care
Source: Infect Dis Rep. 2024 Sep 2;16(5):870–9. doi: 10.3390/idr16050068 (PMC11417937; doi:10.3390/idr16050068)
Supplement: Supplementary file 1 [file idr-16-00068-s001.zip › idr-3088346-supplementary.pdf]

Supplementary Table S1. Study population by year.

Year	No. Patients
2009	1072262
2010	1090157
2011	1098231
2012	1106646
2013	1107404
2014	1108360
2015	1103076
2016	1087788
2017	1064504
2018	1035167
2019	1000365
2020	1186814
2021	1126786
2022	1037693
